# Use of traditional eye medicine and self-medication in rural India: A population-based study

**DOI:** 10.1371/journal.pone.0183461

**Published:** 2017-08-22

**Authors:** Noopur Gupta, Praveen Vashist, Radhika Tandon, Sanjeev K. Gupta, Mani Kalaivani, S. N. Dwivedi

**Affiliations:** 1 Dr. Rajendra Prasad Centre for Ophthalmic Sciences, All India Institute of Medical Sciences, New Delhi, India; 2 Centre for Community Medicine, All India Institute of Medical Sciences, New Delhi, India; 3 Department of Biostatistics, All India Institute of Medical Sciences, New Delhi, India; Aston University, UNITED KINGDOM

## Abstract

**Objective:**

To determine the type and nature of traditional eye medicine (TEM), their sources and use and practices related to self-medication for ophthalmic diseases in a rural Indian population.

**Methods:**

A population-based, cross-sectional study was conducted in 25 randomly selected clusters of Rural Gurgaon, Haryana, India as part of CORE (Cornea Opacity Rural Epidemiological) study. In addition to comprehensive ophthalmic examination, health-seeking behavior and use of self-medication and TEM was assessed in the adult population using a semi-structured questionnaire. Physical verification of available ophthalmic medications in the enumerated households was conducted by the study team. Descriptive statistics were computed along with multivariable logistic regression analysis to determine associated factors for use of self-medication and TEM.

**Results:**

Of the 2160 participants interviewed, 396 (18.2%) reported using ophthalmic medications without consulting an ophthalmologist, mainly for symptoms like watering (37.1%), redness (27.7%), itching (19.2%) and infection (13.6%). On physical verification of available eye drops that were being used without prescription, 26.4% participants were practicing self-medication. Steroid, expired/unlabeled and indigenous eye drops were being used by 151(26.5%), 120(21.1%) and 75 (13.2%) participants respectively. Additionally, 25.7% (529) participants resorted to home remedies like *‘kajal’(61*.*4%)*, honey (31.4%), ghee (11.7%) and rose water (9.1%).

**Conclusion:**

Use of TEM is prevalent in this population. The rampant use of steroid eye drops without prescription along with use of expired or unlabelled eye drops warrants greater emphasis on safe eye care practices in this population. Public awareness and regulatory legislations must be implemented to decrease harmful effects arising due to such practices.

## Introduction

Infectious keratitis contributes significantly to ocular morbidity and corneal blindness, especially in developing nations. [1,2] In India, corneal blindness is the leading cause of blindness after cataract. [3] The indiscriminate use of traditional eye medicines (TEM) in developing countries including India is responsible for increased occurrence of corneal infections and ulcerations in these regions. [4]Traditional eye medicines are biologically derived therapies that are usually dried parts of various plants that are rendered soluble in an aqueous medium. They can also be of animal or human origin and include breast milk, saliva and urine. [5,6]

The development and exacerbation of corneal infections occurring due to these harmful practices, frequently result in poor visual outcome.[7–9]The underlying factors for development of infection has been attributed to contamination and delay in proper anti-microbial therapy due to the use of TEM. [5] They may also act as a carrier of infection and may create a conducive environment for proliferation of pathogenic organisms.[7] Due to their direct harmful and noxious effect, they may cause corneal epithelial breakdown and thus aid in bacterial penetration to deeper corneal layers. Hence, indiscriminate use of traditional eye medicines may account for increased occurrence of infectious keratitis in the developing world.[5–6]

Use of self-administered eye drops for ophthalmic conditions is a common practice in rural populations. [10]The use of self-administered therapy in cases of ophthalmic disease can delay institution of effective therapy and negatively impact visual outcome.[11]It has been reported to be an expected outcome of a malfunctional health care system with poor accessibility to quality eye care services.[10]Attitudes and practices related to self-treatment have not been studied extensively in the Indian population, especially in North India. Hence, this cross-sectional, population-based study was planned to understand the current practices and behaviour of a rural Indian population and to determine the frequency, occurrence and characteristics of use of TEM and self-medication through a population-based, house-to-house survey.

## Methods

The Corneal Opacity Rural Epidemiological (CORE) study was conducted in 25 randomly selected clusters of Rural Gurgaon (renamed ‘Gurugram’ since 2016), state of Haryana, India during 2011–2013, the detailed methodology has been discussed previously.[2] It estimated the burden and causes of corneal diseases through a cross-sectional, population-based study design. The study was approved by the Institutional Ethics Committee, All India Institute of Medical Sciences, New Delhi, India *(Reference number*: *IESC/T-1380/01*.*04*.*2011)*. Approval from the local administrative authorities and ‘*sarpanch’* of respective villages was also sought. The examination and survey protocol was explained to each eligible adult. As per guidelines laid down by Declaration of Helsinki, written informed consent for enrolment, ophthalmic examination and questionnaire administration was taken from all eligible adults.

The training of all members of the study teams was conducted and it included a pre-pilot survey in a village in Faridabad, Haryana. Subsequently, a pilot survey was conducted in a non-study cluster wherein all study-related procedures were followed. A house-to-house visit was made to examine all enumerated individuals in the 25 rural clusters, each comprising of 500–600 people. In addition to comprehensive ophthalmic examination, attitudes and practices related to instillation of self-medication and TEM was assessed through a semi-structured questionnaire. The questionnaire was pre-tested and validated using standard procedures. A team of five experts which included a statistician, public health specialists, social workers and ophthalmologists finalized the questionnaire for the study. Pre-testing of the questionnaire included nearly 120 participants from the hospital, primary eye care clinics and the general community. After this step, redundant and confusing questions were removed, some items were re-framed to improve flow of administration and increase its effectivity in capturing survey data. The survey questionnaire was then finalized and administered in the pilot cluster. A total of 45 participants were included in the pilot survey in the non-study cluster. The questionnaire (S1 File) was then administered to a representative adult population in the 25 study clusters. Thus, the adult population interviewed for use of TEM and self-treatment included both people with and without corneal opacity, as ascertained through clinical examination by the ophthalmologist.

During the house-to-house visits, attitudes and practices related to use of TEM was noted and each study participant was asked about usage of TEM. The use of self-prescribed medications for eye disorders was also enquired. Practices related to self-medication were enquired and the usual symptom for use of ophthalmic medicines without prescription was also recorded. All people in the identified clusters were provided with a unique identification number and their socio-demographic details were noted in the household enumeration form.

Available ophthalmic medications in the households of the eligible participants were physically verified by the study team. The trade name, generic name if available, composition with their constituents & their concentration were noted in detail. The expiry date on the label of the medicine, whenever available, was noted. In the questionnaire, responses were noted verbatim as given by the respondent in the local Hindi language. Additionally, the source of eye-related health information and health-seeking behavior of the study population was noted to interpret the study findings in a holistic manner.

Visual impairment (VI) and blindness in the CORE study was defined as per WHO (World Health Organization) criteria. As per these criteria, VI and blindness was defined as visual acuity of less than 6/18 and less than 3/60 in the better eye, with available correction. Quality assurance and standardization of all study procedures and equipments was maintained all throughout the study.

### Statistical analysis

Microsoft Access based platform with inbuilt validation and consistency checks was utilized for data entry. The collected data was analyzed through Stata 12.0 (Stata, College Station, Texas) software. Qualitative and quantitative data is represented as number (%) and mean ± standard deviation and median (range) respectively. Qualitative response variable were analyzed between the groups using chi-square test/Fisher’s exact test. Univariate and multivariable logistic regression analysis was conducted to find any association between socio-demographic and clinical factors with the practice of self-treatment and traditional eye medicine. The results for the same were reported as Odds Ratio (95% CI). Any probability value of less than 0.05 was considered statistically significant.

## Results

### Demographic characteristics of the study population

In order to record practices related to usage of self-treatment and application of TEM, 2160 adults were interviewed. There were nearly equal proportion of males (n = 1007;46.6%) and females (n = 1153;53.4%) interviewed from the 25 rural clusters. Mean age of participants was 55.6 ±13.2 years and 61.1% (n = 1319) participants were aged 50 years and older. In this interviewed population, nearly half (n = 985;45.6%) of the study participants were involved in housework and 65.7% (n = 1419) people were either illiterate or educated up to primary level only.

### Source of eye-related health information

The most frequently reported source of eye-related health information by the participants were neighbors, relatives and traditional healers (n = 1588; 73.5%). The other sources of eye-related information were hospital & health workers (n = 365; 16.8%), patients (n = 116; 5.6%), newspapers/books/pamphlets (n = 86; 3.9%), audio-visual media like television & radio (n = 53; 2.4%) and school teachers (n = 7; 0.5%). Nearly 7% (n = 67) participants did not seek any health-related information from any source and some acquired such information from multiple sources.

### Utilization of health care services

On enquiring about the choice of health care facility sought by the participants in case of any ophthalmic problem, nearly 14.2% (n = 307) reported that they did not consult an ophthalmologist for eye problems. Out of those seeking advice from a non-ophthalmologist for eye problems, traditional healers, drug stores, non-registered practitioners, pharmacists were approached by 44.7% (138 of 307) and another 45.6% (140 of 307) of them resorted to home remedies and did not visit anybody for ophthalmic problems.

The barriers for not utilizing ophthalmic services in the study population were the distant location of the hospital and health care facilities (n = 80;38.8%), objection raised by older family members (n = 58;28.2%), acceptable services provided by the local healers (n = 53;25.7%), economic constraints (n = 39;18.9%), and for some, ophthalmic disease was not a priority (n = 18;8.7%). Multiple barriers could be reported by a single participant. During the house-to-house survey, 85 participants reported having sustained ocular trauma. It was interesting to note that no treatment was sought in spite of ocular injury by 35 (41.2%) patients and nine (18%) presented to non-ophthalmologists while seven (14%) took treatment from traditional healers, ‘hakim’ (a physician using traditional remedies in India and Muslim countries), ‘vaid’ (a practitioner of ayurvedic system of medicine which is an ancient Hindu science of health and medicine) and other non-registered practitioners. Traditional eye medicine was used by 14 of 85 (16.4%) patients with ocular injury for relief of their symptoms.

### Participant-reported self-treatment

Nearly one-fifth (n = 396;18.3%) of the population interviewed, reported use of ophthalmic medicines without consulting an ophthalmologist for symptoms of watering (n = 147;37.1%), redness (n = 110; 27.7%), itching (n = 76;19.2%), redness with discharge (n = 54;13.6%), painful eyes (n = 49;12.4%), foreign body sensation (n = 40;10.1%), diminution of vision (n = 20;5.1%) and burning of the eyes (n = 15;3.8%). Other participants used eye medicines without prescription for cleaning of eyes, removing foreign bodies from the eyes, ocular discharge and swelling, glare, ocular trauma, tiredness of eyes and for prevention of cataract. This was a multiple response item in the questionnaire and the participants could enumerate more than one reason for using eye drops without ophthalmic consultation. Older participants (p = 0.006), unmarried (p = 0.044)and less educated (p = 0.028) people were more likely to self-medicate (Table 1). On multivariable logistic regression analysis to account for various factors like age, gender, marital status, religion, level of education, occupation, presence of corneal opacity due to infective keratitis or ocular trauma, presence of visual impairment in the study population (Table 1), no significant differences were found in self-medication practices according to age (p = 0.962), gender (p = 0.599), level of education (p = 0.41) or religion (p = 0.632).

**Table 1:** Association of socio-demographic and clinical factors with use of self-medication in the study population.

	Use of self-medication		Unadjusted OR (95% C.I.)		Adjusted OR (95% C.I.)	
Risk factors	No n (%)	Yes n(%)		P value		P value
**Age (years)**						
18–49	703 (83.6)	138 (16.4)	1		1	
50–59	418 (83.9)	80 (16.1)	0.97 (0.72, 1.32)	0.869	0.98 (0.41, 2.37)	0.962
≥60	643 (78.3)	178 (21.7)	1.41 (1.10, 1.80)	0.006*	1.72 (0.77, 3.80)	0.184
**Gender**						
Male	820 (81.4)	187 (18.6)	1		1	
Female	944 (81.9)	209 (18.1)	0.97 (0.78, 1.21)	0.790	0.82 (0.39, 1.71)	0.599
**Marital Status**						
Married	1408 (82.5)	298 (17.5)	1		1	
Unmarried	356 (78.4)	98 (21.6)	1.30 (1.01, 1.68)	0.044*	1.12 (0.62, 2.01)	0.716
**Religion**						
Hindu	1747 (81.8)	390 (18.2)	1		1	
Muslim	17 (73.9)	6 (26.1)	1.58 (0.62, 4.04)	0.338	1.77 (0.17, 18.35)	0.632
**Education**						
Above primary(>Std 5)	624 (84.2)	117 (15.8)	1		1	
Up to primary**	1140 (80.3)	279 (19.7)	1.31 (1.03, 1.65)	0.028*	1.36 (0.65, 2.84)	0.410
**Occupation**						
House work	808 (82.0)	177 (18.0)	1		1	
Agricultural Work	187 (84.2)	35 (15.8)	0.85 (0.58, 1.27)	0.436	1.21 (0.37, 4.00)	0.750
Non-agricultural Work	211 (78.2)	59 (21.8)	1.28 (0.92, 1.78)	0.149	1.80 (0.64, 5.09)	0.266
Indoor Work	201 (82.4)	43 (17.6)	1.01 (0.70, 1.45)	0.972	1.53 (0.48, 4.86)	0.467
Not working	357 (81.3)	82 (18.7)	1.05 (0.78, 1.40)	0.749	1.00 (0.49, 2.05)	0.993
**Infective Keratitis**						
Absent	283 (81.1)	66 (18.9)	1		1	
Present	64 (81.0)	15 (19.0)	1.01 (0.54, 1.87)	0.988	1.00 (0.52, 1.90)	0.989
**Ocular Trauma**						
Absent	276 (81.4)	63 (18.6)	1		1	
Present	71 (79.8)	18 (20.2)	1.11 (0.62, 1.99)	0.725	1.16 (0.63, 2.14)	0.642
**Visual Impairment**						
Absent	124 (83.2)	25 (16.8)	1		1	
Present	223 (79.9)	56 (20.1)	1.25 (0.74, 2.10)	0.408	0.99 (0.51, 1.93)	0.974

OR = Odds Ratio; CI = Confidence Interval

*statistically significant difference between groups

**Includes participants educated up to Class 5 of formal schooling and people who could read and write.

### Magnitude of self-treatment as ascertained by physical verification

To impart objectivity to the study and eliminate recall bias, physical verification of any available medicines in the household of the participants was conducted. A significant number (n = 570; 26.4%) of participants were found to be using eye medicines without prescription. Expired or unlabelled eye drops without any mention of expiry date were found in households of 120 (21.1%) participants. Steroid eye drops and ointments were being used without prescription by 151(26.5%)participants and non-steroidal anti-inflammatory drugs by 73 (12.8%) participants. Other herbal and indigenous eye drops belonging to the alternative medicines were also being commonly used by 75 (13.2%) participants.

### Use of traditional eye medicine

A total of 2055 participants were asked about the use of any traditional product in their eyes as home remedies. Nearly one-fourth (n = 529;25.7%) of the study population resorted to home remedies and reported using some form of traditional eye medicine. The traditional eye medicines commonly used in this study group (n = 529) were ‘*kajal’(*n = 325;*61*.*4%)*, honey (n = 166;31.4%), ghee(n = 62; 11.7%) and rose water (n = 49;9.1%). Some peculiar TEMs being instilled in the eye by this population were alum water (n = 15;2.8%), milk (n = 4;0.8%), plant juices (n = 4;0.8%), saline water (n = 26;4.9%), breast milk (n = 3;0.6%), turmeric (n = 10;1.9%), jaggery (n = 7;1.3%), curd (n = 2;0.4%), garlic (n = 2;0.4%), goat’s milk (n = 3;0.6%), ‘*neem’*(n = 15;2.8%), powdered horn of deer (n = 5;0.9%), excreta of donkey (n = 3;0.6%), lemon juice (n = 5;0.9%), turpentine oil (n = 2;0.4%), coconut oil (n = 3;0.6%), warm tea leaves (n = 3;0.6%), ginger juice (n = 11;2.1%), onion juice (n = 8;1.5%), ash of *hukkah*(n = 5;0.9%), mustard oil (n = 3;0.6%), fenugreek (n = 5;0.9%), carom seeds (*ajwain*) (n = 3;0.6%) and leaf extracts (n = 6;1.1%). Instillation of TEM was reported to be higher in the elderly age group (p = 0.004), Muslims (p = 0.006) and in illiterates (p = 0.001) (Table 2). On applying multivariable logistic regression to account for various factors like age, gender, marital status, religion, level of education, occupation, presence of corneal opacity due to ocular trauma, presence of visual impairment in the study population (Table 2), participants with no visual impairment were more likely to use TEM (p = 0.016).

**Table 2:** Association of socio-demographic and clinical factors with use of traditional eye medicines (TEM) in the study population.

Risk factors	Use of TEM		Unadjusted OR (95% C.I.)		Adjusted OR (95% C.I.)	
	No n(%)	Yes n(%)		P value		P value
**Age (years)**						
18–49	589 (71.8)	231 (28.2)	1		1	
50–59	336 (72.3)	129 (27.7)	0.98 (0.76, 1.26)	0.869	1.45 (0.67, 3.06)	0.330
≥60	601 (78.0)	169 (22.0)	0.72 (0.57, 0.90)	0.004*	1.73 (0.83, 3.60)	0.143
**Gender**						
Male	732 (76.1)	230 (23.9)	1		1	
Female	794 (72.6)	299 (27.4)	1.20 (0.98, 1.46)	0.075	1.49 (0.68, 3.27)	0.322
**Marital Status**						
Married	1199 (73.8)	425 (26.2)	1		1	
Unmarried	327 (75.9)	104 (24.1)	0.90 (0.70, 1.15)	0.389	0.71 (0.38, 1.31)	0.276
**Religion **						
Hindu	1515 (74.6)	517 (25.4)	1		1	
Muslim	11 (47.8)	12 (52.2)	3.20 (1.40, 7.29)	0.006*	0.84 (0.08, 8.78)	0.888
**Education**						
Above primary (>5^th^Std)	566 (78.7)	153 (21.3)	1		1	
Up to primary**	960 (71.9)	376 (28.1)	1.45 (1.17, 1.80)	0.001*	1.01 (0.50, 2.03)	0.980
**Occupation**						
House work	663 (71.5)	264 (28.5)	1		1	
Agricultural Work	160 (75.1)	53 (24.9)	0.83 (0.59, 1.17)	0.291	1.03 (0.32, 3.33)	0.957
Non-agricultural Work	178 (68.5)	82 (31.5)	1.16 (0.86, 1.56)	0.338	1.62 (0.58, 4.56)	0.358
Indoor Work	176 (74.6)	60 (25.4)	0.86 (0.62, 1.19)	0.350	1.30 (0.43, 3.92)	0.637
Not working	349 (83.3)	70 (16.7)	0.50 (0.38, 0.68)	<0.001*	0.81 (0.39, 1.68)	0.567
**Infective Keratitis**						
Absent	251 (77.0)	75 (23.0)	1		1	
Present	57 (77.0)	17 (23.0)	1.00 (0.55, 1.82)	0.995	1.20 (0.63, 2.26)	0.581
**Ocular Trauma**						
Absent	251 (77.9)	71 (22.1)	1		1	
Present	57 (73.1)	21 (26.9)	1.30 (0.74, 2.29)	0.360	1.36 (0.75, 2.47)	0.319
**Visual Impairment**						
Absent	98 (69.0)	44 (31.0)	1		1	
Present	210 (81.4)	48 (18.6)	0.51 (0.32, 0.82)	0.005*	0.47 (0.25, 0.87)	0.016*

OR = Odds Ratio; CI = Confidence Interval; TEM = Tradition Eye Medicine

*statistically significant difference between groups

**Includes participants educated up to Class 5 of formal schooling and people who could read and write.

## Discussion

Use of traditional eye medicine is recognized as an important contributory factor for development as well as for delayed or complicated presentation of corneal ulcer cases, especially in rural populations.[6,12]To the best of our knowledge, there has been no population-based evaluation of the use of traditional eye medicines and self-medication through physical verification of self-administered eye drops, worldwide. Our study highlights that the rural Indian population utilizes various traditional, home-based remedies and self-prescribed ophthalmic medications for relief of ocular symptoms without accessing the health care system. Self-medication with ophthalmic steroids and use of expired & unlabeled drops was prevalent in the study population.

Traditional eye medicine was commonly used (25.7%) in this rural population in the form of ‘*surma/kajal’*, honey, ghee, rose water and other plant, dairy and animal products. Likewise, a hospital-based study from Sao Paulo, Brazil reported use of homemade, traditional products like boric acid, normal saline and herbal infusions for ophthalmic emergencies.[11] It is also important to note that TEM was being applied more frequently by people who sustained ocular trauma in our study population, hence there is a need to create awareness and impart health education amongst the people about primary eye care and early referral of such cases.

Use of traditional eye medicines and self-medication has been documented in a variety of settings, especially in people of rural residence. [12] There is limited information on community-based practices related to use of traditional medicine, especially traditional eye medicine.[13]Our reported prevalence of use of TEM was higher as compared to a hospital-based study conducted in the Nigerian population where only 5.9% patients were using TEM. [14]The reported prevalence of use of TEM in other hospital-based studies in the same region was 1.5% and 13.2%. [15,16] Our study suggests that traditional eye practices are being widely used by the Indian population and is not dependent on the participants’ age, gender, level of education, religion or marital status. Similar results were reported from Sao Paulo in Brazil.[11]To date, available studies on use of TEM in normal subjects are either hospital-based or have mostly been done in the African countries.[12,14–17] As population-based studies provide more accurate data on TEM use, this study reflects the actual burden of use of TEM in this rural North Indian population.

In our study population, use of ophthalmic medicines without prescription was reported by nearly one-fifth (18.4%) of the population. Similar rates of self-treatment have been reported from rural Malawi in Africa and Campinas in Brazil [10,18]Hospital-based studies from Latin America also report a high usage of self-medication (25.6% in Argentina, 25.7% in Columbia and 75% in Southern Chile) where non-steroidal anti-inflammatory eye drops were the most common drug used. [19–21] On the contrary, antibiotics were the most common ophthalmic drug used for self-treatment in United Arab Emirates.[22] The difference in the magnitude of self-treatment is also attributed to differences in study design. The study from Chile reports greater magnitude as all kinds of over-the-counter medicines bought from the pharmacy were taken into account and was not restricted to ophthalmic medications.[21]

On physical verification of available medicines in the households, the usage of self-treatment rose to 26.4% in our study population. As expired or unlabelled eye drops were being used by a significant number of participants in this rural population, public awareness and regulatory legislations must be implemented to decrease the harmful effects arising due to such practices. This has also been reiterated in another study from India where people using self-treatment, were not aware of the components or expiry date of self-administered ophthalmic preparations.[23]The rampant use of self-prescribed steroid and non-steroidal anti-inflammatory eye drops should be curtailed in order to prevent development of non-healing corneal ulcers. Simple medications like non-preserved tear substitutes may be made available for routine use in pharmacies or drugstores.[24] But, caution needs to be taken in use of ayurvedic and indigenous eye drops, which are available off-the-counter without consultation of an ophthalmologist.

The commonest sources of eye-related health information in this study population were villagers themselves that included neighbors, relatives and traditional healers. People residing in villages and rural parts of the developing world, usually tend to consult local healers or elders of the community in the event of an ocular disease as they believe that diseases are caused by violating traditional societal rules [12] Hence, large-scale efforts at public education need to be evolved and implemented through ground-level health care volunteers and local community participation. There are no previous Indian studies which clearly exemplify the common sources of eye-related health information in the general population. In the Nigerian population, mass media was the source of eye-related health information in 53.3%, relatives or family members in 20% and eye specialists in 13.3%. [25]There is a need to improve health literacy in similar populations to resolve health-related barriers, understand cultural habits and avoid harmful traditional practices.

In the present study, 8.4% of the participants consulted a non-ophthalmologist for eye problems. Traditional healers, drug stores, non-registered practitioners, pharmacist and chemists were approached by people for relief of their ophthalmic problems. The barriers for not utilizing ophthalmic services were the distant location of the hospital and health care facilities, economic constraints, objection raised by other members of the family, satisfactory treatment provided by traditional healers, ophthalmic disease not being a priority and some resorted to home-made remedies and thus did not take any ophthalmic advice. Similar barriers have been reported by previous studies. [19,20, 26–28]There is a need to eliminate these barriers by generating awareness through educational campaigns and to improve accessibility, affordability and availability of quality ophthalmic services for the general population. It is imperative to conduct qualitative research studies in order to understand peoples’ behaviour in different populations to combat the current problem.It is also important to strengthen the community eye care referral network and develop locally acceptable plans with a goal to eliminate avoidable corneal blindness.

Our study has a few limitations. The age distribution of the interviewed population reveals that the proportion of older age group was more than the younger adults (aged 18–40 years). This is because this age group (18–40 years) comprises of the productive population and was out at work when the house-to-house visits were conducted. Thus, the increased prevalence of TEM in our population could be the result of a higher proportion of older participants. Additionally, we recommend that assessment of use of TEM and self-medication should be conducted amongst urban populations in order to provide a holistic picture on use of TEM in the Indian community.

Our study is unique as it combines detailed clinical examination of all participants who were interviewed about practices related to use of self-medication and traditional eye medicine. It highlights the need for effective execution and establishment of accessible, high-quality primary eye care services and an organized, healthcare referral network. To reverse the trend and decrease the use of TEM and its consequences, we need to strengthen eye care programmes and enhance delivery of promotive, preventive and curative healthcare to such underserved rural populations.

## Supporting information

S1 File Questionnaire used for the study. (PDF)
