# Supplementary material for: Use of traditional eye medicine and self-medication in rural India: A population-based study
Source: PLoS One. 2017 Aug 22;12(8):e0183461. doi: 10.1371/journal.pone.0183461 (PMC5567472; doi:10.1371/journal.pone.0183461)
Supplement: S1 File — (PDF) [file pone.0183461.s001.pdf]

कार्निया फार्म –IV

कार्नियल अस्पष्टता संबंधी समुदाय आधारित एपिडिमियोलाजिकल अध्ययन

IDENTIFICATION DATA<br>पहचान तथ्य	RESPONSE CATEGORIES<br>उत्तरदाता श्रेणी	FOR OFFICE USE ONLY<br>केवल कार्यालय हेतु
Name of Village<br>ग्राम का नाम	____________________	[ ] [ ]
Cluster Code<br>समूह संख्या	____________________	[ ] [ ]
Household ID<br>मकान को दिया गया नम्बर	**Study House No.**___________________	[ ] [ ] [ ] [ ] [ ] [ ]<br>Location ID (1) + Cluster ID (2)+ HH No. (3)<br>जगह संख्या (1) + समूह संख्या (2)+ मकान संख्या (3)
Interviewer Name and Code<br>साक्षात्कारकर्ता का नाम एवं कोड	[ ] [ ]	[ ] [ ]
Respondent Name<br>उत्तरदाता का नाम		
Person Unique ID No.<br>**(From Enumeration Form)**<br>व्यक्ति को दिया गया नम्बर	Person Number.____________<br>व्यक्ति का नम्बर ____________	[ ] [ ] [ ] [ ] [ ] [ ] [ ] [ ]<br>Location ID (1)+ Cluster ID (2)+ HH No (3) + Person No(2)<br>जगह संख्या (1) + समूह संख्या (2)+ मकान संख्या (3) + सदस्य संख्या (2)
Gender<br>लिंग	Male=1; Female=2<br>पुरूष = 1; स्त्री = 2	[ ]
Age<br>उम्र	________ (In completed years)<br>(निकटतम वर्ष में)	[ ] [ ]
Date of Interview<br>साक्षात्कार की तिथि	**(dd/mm/yyyy)** _ _/_ _/_ _ _ _	[ ] [ ]-[ ] [ ] - [ ][ ][ ][ ]

आँखों की बिमारी संबंधित जानकारी, स्वभाव एवं व्यवहार का मूल्यांकन			
**S.No.**	**Question**	**Response**	**Marking Guide**
**1.**	What are the common symptoms of eye diseases?<br>**(Multiple response possible)**<br><br>आप के अनुसार आँखों की बिमारियों के क्या–क्या लक्षण हो सकते है ?<br>(**एक से अधिक उत्तर संभव है**)		a. सर दर्द<br>b. आँखों का लाल होना<br>c. आँखों से पानी आना<br>d. आँखों से कीचड़ आना<br>e. आँखों में जलन<br>f. खुजली होना<br>g. आँखों में किरकिरापन/रगड़न होना<br>h. आँखों में दर्द होना<br>i. धुंधलापन<br>j. अन्य<br>k. नहीं मालूम
**2.**	Which eye diseases have you heard of?<br>**(Multiple response possible)**<br><br>आपने आँखों की कौन –कौन सी बिमारियों के बारे में सुना हैं?<br>**(एक से अधिक उत्तर संभव है)**		a. आईफ्लु (Eye flu), संक्रमण<br>b. दृष्टिदोष<br>c. मोतियाबिन्द<br>d. रोहे/कुर्कुरे/परबाल<br>e. काला मोतिया<br>f. भेंगापन<br>g. फूला<br>h. रतौंधी<br>i. विटामिन ‘A’ की कमी (विटामिन ‘A’ डिफिसिएन्सी)<br>j. नखूना<br>k. अन्य<br>l. नहीं मालूम

**3.**	Have you heard about the following eye diseases?<br>क्या आप ने आँखों की इन बिमारियों के बारे में सुना है?<br><br>**हाँ** **नहीं**<br><br>a. आईफ्लु (Eye flu), संक्रमण ☐ ☐<br>b. दृष्टिदोष ☐ ☐<br>c. मोतियाबिन्द ☐ ☐<br>d. रोहे/कुर्कुरे/परबाल ☐ ☐<br>e. काला मोतिया ☐ ☐<br>f. भेंगापन ☐ ☐<br>g. फूला ☐ ☐<br>h. आँख की पारदर्शी झिल्ली पर सफेद दाग (Corneal Opacity) ☐ ☐<br>i. रतौंधी ☐ ☐<br>j. विटामिन 'A' की कमी (विटामिन 'A' डिफिसिएन्सी) ☐ ☐<br>k. नखूना ☐ ☐		
**4.**	What is the source of information about eye disease to you and your family members?<br>**(Multiple response possible)**<br>आपको एवं आपके परिवारजनों को आँखो की बिमारी संबंधी जानकारी कहाँ से मिलती है?<br>(**एक से अधिक उत्तर संभव है**)		a. स्वास्थ्य कार्यकर्त्ता<br>b. टेलीविजन<br>c. समाचार पत्रो द्वारा/पम्पलेटस (Pamphlets)<br>d. रेडियो (Radio)<br>e. पड़ोसी<br>f. रिश्तेदार/घर का सदस्य<br>g. अन्य<br>h. कहीं से नही मिलती

**5.**	If case of any eye problem, whom do you consult first?<br>**(Multiple response possible)**<br>(If answer 'a' or 'b' in no then go to QNo. 9)<br><br>आँख की समस्या होने पर आप पहले कहाँ जाते है?<br>**(एक से अधिक उत्तर संभव है)**<br>(यदि उत्तर '**a**' या '**b**' है तो प्रश्न सं० **7** पर जायें)		a. निजी नेत्र विशेषज्ञ<br>b. सरकारी अस्पताल के नेत्र विशेषज्ञ के पास<br>c. सामान्य डाक्टर/स्थानीय चिकित्सक<br>d. सरकारी डिस्पेन्सरी/प्राथमिक स्वास्थ्य केन्द्र<br>e. हकीम/वैद्य/गैर पंजीकृत चिकित्सक<br>f. दवा की दुकान<br>g. अन्य
**6.**	In case you do not go to an eye doctor for your eye problems, what are the various reasons?<br>**(Multiple response possible)**<br>यदि आप या आपके परिवारजन नेत्र चिकत्सक को नहीं दिखाते है तो नेत्र चिकित्सक के पास नहीं पहुँचने के क्या–क्या कारण है?(**एक से अधिक उत्तर संभव है**)		a. नेत्र चिकित्सक के विषय में अज्ञानता<br>b. आँख का स्वास्थ्य महत्वपूर्ण नही है<br>c. अस्पताल जाने में समय बहुत लगता है<br>d. इलाज से डर लगता है<br>e. स्थानीय चिकित्सक का उपचार संतोषजनक है<br>f. आर्थिक कारण<br>g. परिवारिक कारण<br>h. अन्य
**7.**	Have you ever used eye medicine on your own without consulting the doctor?<br>(If answer is 'No' then go to Q.No.-9)<br><br>क्या आपने ने कभी नेत्र चिकित्सक के सलाह के बिना, आँखो की दवाई ली है?<br>(यदि उत्तर 'नहीं' है तो प्रश्न सं० **9** पर जायें)	हाँ ☐ नहीं ☐	
**8.**	If "Yes" then for which eye problems, do you use eye medicine on your own without consulting the doctor?<br>**(Multiple response possible)**<br><br>यदि हाँ तो आँख की कौन-कौन सी समस्या के लिये?<br>(**एक से अधिक उत्तर संभव है**)		a. आँखों का लाल होना<br>b. आँखों से पानी आना<br>c. आँख में किरकिरी पड़ना<br>d. नजर कमजोर होना<br>e. आँखों में दर्द<br>f. आँखों में जलन<br>g. आँखों में खुजली<br>h. आँखों की सफाई के लिए<br>i. मोतिया रोकने के लिए<br>j. आँखों का संक्रमण (conjunctivitis<br>k. अन्य

**9.**	Is there any eye medicine available in your house?<br>**(Multiple response possible)**<br>(If answer is 'No' then to Q.No.-11)<br><br>क्या आप के घर में आँख संबंधी कोई दवा उपलब्ध है?<br>(**एक से अधिक उत्तर संभव है**)<br>(यदि उत्तर 'नहीं' है तो प्रश्न सं० 11 पर जायें)		a. दवाई नही है/मान्य नहीं है (Not Applicable)<br>b. Steroid<br>c. गुलाब जल<br>d. अन्य
**10.**	What is the expiry date of the medicine?<br>दवा की Expiry Date क्या है ?	______________________	a. दवा Expired है<br>b. दवा Expired नही है<br>c. दवा की Expiry Date उपलब्ध नही है
**11.**	In case of eye problems, do you apply anything beyond the eye medicine (TEM)? **(Multiple response possible)**<br><br>आँख की समस्या होने पर, क्या आप दवा के अतिरिक्त कुछ और इस्तेमाल करते है ? (उल्लेख करें)<br>(**एक से अधिक उत्तर संभव है**)		a. दुध<br>b. नारियल का तेल<br>c. घी<br>d. शहद<br>e. काजल/सुरमा<br>f. हल्दी<br>g. नीम/नीम का लेप<br>h. अन्य<br>i. कुछ नहीं
**12.**	In case of trauma to the eye, when do you think you should seek advice from the eye doctor?<br>यदि आँख में चोट लगती है, तो आप के अनुसार नेत्र चिकित्सक की सलाह कितने समय में लेनी चाहिए ?	______________________	a. तुरन्त<br>b. 0-6 घंटे<br>c. 7-24 घंटे<br>d. 1-7 दिन<br>e. किसी भी समय<br>f. आवश्यकतानुसार<br>g. नहीं मालूम
**13.**	Can the following things cause of injury to the eye?<br>क्या इन वस्तुओं से आँखों को नुकसान हो सकता है ?		

Item	हाँ	नहीं	नहीं मालूम
a. आँख में गुलाब जल डालने से (Gulabjal)	☐	☐	☐
b. आँख में चूना गिरने से (Chuna injury)	☐	☐	☐
c. आँख में होली के रंग गिरने से (Holi colors)	☐	☐	☐
d. पटाखे की चोट से (Fire cracker injury)	☐	☐	☐
e. आँखों में तेजाब, फिनाईल पड़ने से (Acid & Phenyl injury)	☐	☐	☐

**14.**	In case of minor trauma with vegetative matter, do you consult an eye doctor?<br>फसल/खेत संबंधी कार्य करते समय अगर आँख में छोटी चोट या खरोंच लगती है तो क्या आप डाक्टर से संपर्क करते है?		a. हाँ<br>b. नहीं<br>c. फसल/खेत संबंधी कार्य नही करते है
**15.**	Do you know that deficiency of Vitamin 'A' mostly affects which age group?<br><br>क्या आप जानते है, विटामिन '**A**' की कमी किस उम्र को अधिकतर प्रभावित करती है?	________________	a. 0-6 वर्ष में<br>b. गर्भवती महिलाओं में<br>c. अन्य<br>d. नहीं मालूम
**16.**	Can you tell us about the sources of vitamin 'A' in our diet?<br><br>हमारे आहार में विटामिन 'A' के क्या–क्या स्रोत है?		a. हरी पत्तेदार सब्जीयाँ<br>b. पीले और नारंगी फल जैसे –पपीता, आम, क्द्दू<br>c. दुध<br>d. दाल<br>e. अंडा<br>f. आलू<br>g. मांस/मछली<br>h. अन्य<br>i. नहीं मालूम
**17.**	When is the immunization for measles administered?<br>खसरे का टीका कब लगाया जाता है?		a. 0-9 महीने में<br>b. 10-12 महीने में<br>c. 1-5 वर्ष में<br>d. अन्य<br>e. नहीं मालूम
**18.**	Have you ever heard about eye donation?<br>**(If answer is 'No', then interview is over)**<br><br>क्या आपने कभी आँख दान (नेत्रदान) के बारे में सुना है?<br>(**यदि उत्तर 'नहीं' है तो आगे के प्रश्नों को नहीं पूछें, साक्षत्कार समाप्त करें**)	हाँ ☐ नहीं ☐	

**19.**	When should eyes be donated? (If answer is not 'b' then go to QNo. 21)<br><br>क्या आप जानते हैं कि आँख दान कब किया जाता है? (उल्लेख करें)<br>(यदि उत्तर '**b**' नहीं है तो प्रश्न सं० **21** पर जाये)	______________________	a. मृत्यु के पहले<br>b. मृत्यु के बाद<br>c. किसी भी उम्र में<br>d. अन्य<br>e. नहीं मालूम
**20.**	Do you know that till what time after death can eyes be donated and used?<br><br>क्या आप जानते हैं कि मृत्यु के बाद आँख दान कितने समय के अन्दर करनी चाहिये?	______________________	a. 0-6 घंटे में<br>b. 7-24 घंटे में<br>c. 24 घंटे से अधिक समय<br>d. कभी भी<br>e. नही मालूम
**21.**	According to you, why don't people donate their eyes?<br>**(Multiple response possible)**<br><br>आप के अनुसार लोग, आँख दान क्यों नही करते है?<br>(**एक से अधिक उत्तर संभव हैं**)		a. अज्ञानता<br>b. रिश्तेदारों द्वारा आपत्ति<br>c. समय का अभाव<br>d. धार्मिक मान्यता<br>e. शरीर की बनावट खराब हो जाती है/शरीर से आँखों को अलग करना अस्वीकार<br>f. कानूनी समस्या<br>g. आँख दान करने से मरने वाले की आत्मा को शान्ति नही मिलती<br>h. आँखें या शरीर अस्वस्थ्य होने की वजह से<br>i. अन्य<br>j. नहीं मालूम
**22.**	Do you know what kind of blindness can be corrected by corneal transplantation?<br><br>क्या आप जानते है कि आँख दान के द्वारा किस प्रकार की अंधता को दूर किया जा सकता है?		a. पुतली पर सफेद दाग<br>b. काला मोतिया<br>c. सफेद मोतिया<br>d. आँख के परदे की बीमारी<br>e. जन्मजात अंधापन<br>f. अन्य<br>g. नहीं मालूम

Status of Interview<br><br>साक्षात्कार की स्थिति	1.Completed interview (पूर्ण साक्षात्कार)<br><br>2.Incomplete interview (अपूर्ण साक्षात्कार)<br><br>66.Others (Specify) अन्य (उल्लेख करें)_________

Signature of Interviewer:

Name of Interviewer:
